# Temporal dynamics of depressive symptoms and cognitive decline in the oldest old: dynamic time warp analysis of the Leiden 85-plus study

**DOI:** 10.1093/ageing/afae130

**Published:** 2024-07-02

**Authors:** Abe J C van der Slot, Anne Suzanne Bertens, Stella Trompet, Simon P Mooijaart, Jacobijn Gussekloo, Frederiek van den Bos, Erik J Giltay

**Affiliations:** Department of Psychiatry, Leiden University Medical Center, Leiden, The Netherlands; Department of Psychiatry, Leiden University Medical Center, Leiden, The Netherlands; Mental Health Care Rivierduinen, Old Age Psychiatry Outpatient Clinic, Leiden, The Netherlands; Department of Internal Medicine, Section of Gerontology and Geriatrics, Leiden University Medical Center 2333 ZA Leiden, The Netherlands; LUMC Center for Medicine for Older People, Leiden University Medical Center, Leiden, The Netherlands; Department of Internal Medicine, Section of Gerontology and Geriatrics, Leiden University Medical Center 2333 ZA Leiden, The Netherlands; LUMC Center for Medicine for Older People, Leiden University Medical Center, Leiden, The Netherlands; Department of Internal Medicine, Section of Gerontology and Geriatrics, Leiden University Medical Center 2333 ZA Leiden, The Netherlands; LUMC Center for Medicine for Older People, Leiden University Medical Center, Leiden, The Netherlands; Department of Internal Medicine, Section of Gerontology and Geriatrics, Leiden University Medical Center 2333 ZA Leiden, The Netherlands; LUMC Center for Medicine for Older People, Leiden University Medical Center, Leiden, The Netherlands; Department of Psychiatry, Leiden University Medical Center, Leiden, The Netherlands; Health Campus The Hague, Department of Public Health and Primary Care, Leiden University Medical Center, The Hague, The Netherlands

**Keywords:** cognitive impairment, depression, oldest old, network analysis, time series, older people

## Abstract

**Background:**

The prevalence of depressive symptoms and cognitive decline increases with age. We investigated their temporal dynamics in individuals aged 85 and older across a 5-year follow-up period.

**Methods:**

Participants were selected from the Leiden 85-plus study and were eligible if at least three follow-up measurements were available (325 of 599 participants). Depressive symptoms were assessed at baseline and at yearly assessments during a follow-up period of up to 5 years, using the 15-item Geriatric Depression Scale (GDS-15). Cognitive decline was measured through various tests, including the Mini Mental State Exam, Stroop test, Letter Digit Coding test and immediate and delayed recall. A novel method, dynamic time warping analysis, was employed to model their temporal dynamics within individuals, in undirected and directed time-lag analyses, to ascertain whether depressive symptoms precede cognitive decline in group-level aggregated results or vice versa.

**Results:**

The 325 participants were all 85 years of age at baseline; 68% were female, and 45% received intermediate to higher education. Depressive symptoms and cognitive functioning significantly covaried in time, and directed analyses showed that depressive symptoms preceded most of the constituents of cognitive impairment in the oldest old. Of the GDS-15 symptoms, those with the strongest outstrength, indicating changes in these symptoms preceded subsequent changes in other symptoms, were worthlessness, hopelessness, low happiness, dropping activities/interests, and low satisfaction with life (all *P*’s < 0.01).

**Conclusion:**

Depressive symptoms preceded cognitive impairment in a population based sample of the oldest old.

## Key points

- Dynamic time warping (DTW) analysis of time series data showed that depressive symptoms preceded cognitive impairment.
- DTW quantifies individual symptom dynamics before these data are aggregated.
- The dynamics of apathy symptoms cluster separately from other mood symptoms on the 15-item Geriatric Depression Scale (GDS-15).

## Introduction

Both depression and cognitive impairment are highly prevalent in older persons [1, 2] and greatly impact quality of life (QoL) and overall well-being [3, 4]. Numerous studies have investigated the relationship between depression and cognitive decline, yielding diverse findings. A link between depression (often at baseline) and subsequent cognitive decline was found in several prospective longitudinal studies [5–7] as well as a meta-analysis [8]. Studies examining the trajectories of depressive symptoms found the ascending and, to a lesser extent, the consistently high trajectory to predict cognitive decline or dementia at follow-up [9, 10]. Conversely, other studies found no longitudinal association between depressive symptoms and cognitive decline [11] or found poor memory function or attention to be prospectively associated with the development of depressed mood [12, 13]. While most prior studies focused on the diagnosis of dementia, we aimed to analyse the nuanced ways in which depressive symptoms and cognitive functions interact over time within individuals.

Traditional linear models often struggle to capture the complex dynamics and causal interactions underlying complex processes. However, by employing a network framework, we can comprehensively evaluate the interdependencies and directional influences among variables. A novel method for this investigation is dynamic time warping (DTW), a nonlinear alignment technique renowned for its ability to capture temporal patterns and quantify shape-based similarities between time-series data [14, 15]. Although extensively employed in other research domains [16, 17], DTW remains relatively underutilised in somatic and psychiatric research, especially in geriatric populations [18, 19]. Thus, our integration of DTW into the analysis represents a novel contribution to the field, offering a unique methodology for time series data and its insights previously unexplored in this context. The unique advantage of DTW lies in its ability to overcome irregularities and temporal distortions that can occur in the context of depressive symptoms and cognitive decline, given its consideration of multiple time points for comparison. Moreover, DTW facilitates the clustering of individual symptoms based on shared temporal characteristics that are first analysed within each individual, after which data are aggregated. This is important as pooled average effects across individuals are not necessarily a reliable estimate of the average outcome for individuals over time (i.e. in case of non-ergodic data) [20]. Consequently, the application of DTW in this context holds significant value for investigating dynamic associations and potential temporal relationships [21].

The Leiden 85-plus study offers a unique opportunity to investigate the temporal relation between sequential assessments of depressive symptoms and cognitive decline in a well-characterised population of individuals aged 85 and older. The primary research question of this study was to determine whether there is a temporal relationship between depressive symptoms and cognitive decline in older people in the community. Based on existing literature, we hypothesised that depressive symptoms would precede subsequent cognitive decline in older people.

## Methods

### Study population

#### Leiden 85-plus study

The Leiden 85-plus study is a prospective cohort study investigating the health, functioning and well-being of the oldest old population during 5 years of follow-up. This observational population-based study focused on individuals residing in Leiden, The Netherlands, who reached the age of 85 between 1 September 1997 and 1 September 1999. Details regarding the study design, methodology and information on missing follow-up data have been published elsewhere [22, 23]. There were no selection criteria in terms of demographic features or health status. With an overall response rate of 87.0%, a total of 599 participants (397 women and 202 men) were included. Medical information was obtained from general practitioner records, pharmacy records and/or nursing home physician records. Participants were included in the current study sample if both cognitive assessments and measurements of depressive symptoms were performed. This was not the case for 97 (35.4%) participants with MMSE <19 points to whom the GDS-15, measuring depressive symptoms, was not administered. Those participants with complete measurements at least three different time points were included, which yielded 325 (54.3%) of 599 participants for the current analysis. Informed consent was obtained from all participants, with consent acquired from guardians for individuals with more severe cognitive impairment. The study received approval from the Medical Ethics Committee of the Leiden University Medical Center with code P1.150.

### Measurements

#### Cognitive function

Cognitive function was assessed using several validated tests in the study at each yearly wave.

(1) The Mini-Mental State Examination (MMSE) was used as a measure of overall cognitive function [24].The total MMSE score ranges from 0 through 30, with lower scores indicating worse cognitive function (see Supplementary Materials, p. 12).
(2) Selective attention and executive function were evaluated using the Stroop-Colour-Word Test Part III [25]. Performance on this test was measured in terms of the time taken to complete the task, with shorter completion times indicating better performance.
(3) Processing speed was assessed using the Letter-Digit Coding Test (LDT), a modification of the Symbol Digits Modality Test [26]. The outcome of this test was determined by the number of correct entries made by participants within a 60-second time frame with lower scores indicating worse cognitive function.
(4) The Picture Word Learning Test (PWLT) involved the successive presentation of 12 pictures at a rate of one picture every 2 seconds [27]. The number of correctly recalled pictures immediately afterwards and after 20 minutes serves as a measure of immediate and delayed recall. A lower score indicated worse cognitive function.

#### Depressive symptoms

Depressive symptoms were assessed using the 15-item Geriatric Depression Scale (GDS) (see Supplementary Materials, p. 13) [28]. The GDS-15 is a commonly used self-report measure specifically designed for older adults. The time span is the past week, and it uses a binary response format (yes/no). The scoring ranges from 0 to 15, with higher scores indicating a higher level of depressive symptoms. The 15-item GDS-15 has demonstrated satisfactory internal consistency reliability (Cronbach’s α ≥ 0.70) across diverse older adult populations [29].

#### Statistical analysis

Sociodemographic and clinical variables at baseline are summarised as means and standard deviations (SD), median with interquartile range (IQR) or numbers with percentages, as appropriate.

**Figure 1:**
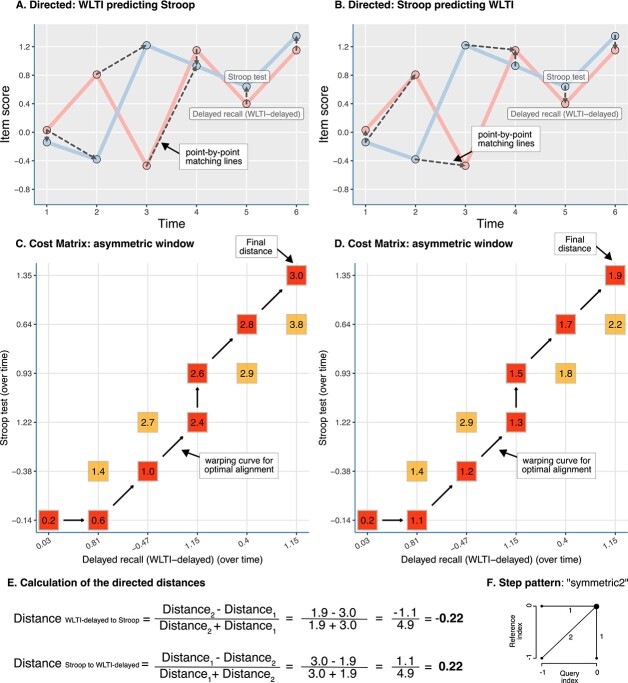
Dynamic time warp direct analysis explanation. The directed dynamic time warp (DTW) analysis is a computational method used to measure the similarity between two time series, in this example, the (standardised) WLTI-delayed scores and their predictive relationship with the Stroop score over time. DTW starts by creating a local cost matrix (LCM) with dimensions corresponding to the lengths of the time series being compared, here being a 6 × 6 matrix as shown in panels (C) and (D). Second, DTW aims to find the optimal path that minimises the total alignment cost between the two series. This process begins at the bottom left corner of the LCM (at LCM [1]) and progresses iteratively to the top right corner (at LCM [6]). During this journey, the algorithm continuously calculates the cumulative distance, known as the ‘cost’. At each step, DTW chooses the path that adds the smallest possible cost, adhering to the constraints set by the chosen algorithm parameters. In this scenario, the constraints are the Sakoe-Chiba window of size 1 and the ‘symmetric2’ step-pattern to determine the path. Panels (A) and (B) illustrate the warping between the time series of the standardised scores of the WLTI-delayed scores and the Stroop score, utilising the asymmetric time window. Stretching is only permitted in one direction, following the current assessment. Panel (E) shows the computation of the directed distance, which is obtained by dividing the difference in distance by the sum of both distances for this variable pair. In this specific example, the direction from the Stroop score to the WLTI-delayed scores yields a value of 0.22. This indicates that alterations in the blue symptom tended to precede changes in the pink item in this particular scenario.

To assess the similarity of variable dynamics over time within participants, we utilised DTW using the panel data [15, 18]. DTW is a technique used to compare and align time series data (Figure 1). DTW works by finding the optimal alignment or warping path between two time series by considering the similarity between corresponding points. Instead of relying on a simple point-to-point comparison, DTW allows for local shifts and deformations in the time axis, enabling a more flexible alignment (i.e. warping). In other words, DTW can be interpreted as a statistical method for evaluating the similarity of two time-series data, searching for similarities between two similarly varying data, even if they are in a slightly different phase. The process involves calculating a ‘cost matrix’ that represents the dissimilarity between each point in one time series and every point in the other. This cost matrix is then used to find the optimal path that minimises the total accumulated distance. In our context, DTW was used to compare variable scores for their trajectories over time to understand which scores tend to go up and down together (in undirected analyses), and which changes precede similar changes in other variables (in directed analyses). Before conducting the analyses, all item scores were *z*-normalised. A stringent *P*-value threshold of <.001 was set for statistical significance, to account for multiple tests. For a detailed explanation of both methods see Supplementary Materials pages 10–11. The correlations between the different items are displayed in a density plot (see Supplementary Materials, p. 9). In order to facilitate comprehension and reproducibility of our approach, we have also provided a short sample R-script (https://osf.io/wx8bk and Supplementary Materials, pp. 4–8).

## Results

### Patient characteristics

Table 1 shows the sociodemographic and clinical characteristics of the included persons at baseline. The total sample consisted of 325 older participants, of whom 68.0% were females and 45.0% had completed intermediate to higher education. At baseline, the median MMSE total score was 27 (IQR = 26–29), the median GDS-15 total score was 1.0 (IQR = 0–3) and 23 (6.8%) of participants had GDS-15 scores of 5 or higher, indicating a possible depression [30]. The included group (*n* = 325) had a significantly higher level of education (*P* < .001), had significantly higher average income (*P* < .001), and were significantly less likely to be institutionalised (*P* < .001) compared with the excluded group. Finally, since dementia at baseline was an exclusion criterion, results show all the cognitive scores at baseline to be significantly higher in the included group compared with the excluded group (*P* < .001).

**Table 1:** Baseline characteristics of the oldest old (aged 85) who were included and excluded in the analyses.

	Entire study population (*n* = 599)	Current study		*P*-value^*^
		Included (*n* = 325)	Excluded (*n* = 274)	
**Socio-demographics**				
Female sex (%)	397 (66.3%)	221 (68.0%)	176 (64.2%)	.34
Low level of education (%)	387 (65.2%)	179 (55.1%)	208 (77.3%)	<.001
Mean income (SD)	934 (487)	1007 (569)	839 (331)	<.001
Institutionalized (%)	107 (17.9%)	27 (8.3%)	80 (29.2%)	<.001
**Comorbidity**				
Diabetes mellitus (%)	86 (14.5%)	38 (11.7%)	48 (17.9%)	.036
Parkinson (%)	16 (2.7%)	5 (1.5%)	11 (4.1%)	.075
Malignancy (%)	104 (17.4%)	56 (17.2%)	48 (17.5%)	.175
**Medication use**				
Antidepressant use (%)	33 (5.5%)	14 (4.3%)	19 (6.9%)	.208
**Functional scores**				
MMSE (IQR)	27.0 (24–28)	27.0 (26–29)	25.0 (22–28)	<.001
GDS-15 (IQR)	2.0 (1–3)	1.0 (0–3)	2.0 (1–4)	<.001
ADL	12.8 (6.3)	10.6 (2.8)	15.5 (8.1)	<.001
IADL	20.1 (8.8)	16.4 (6.4)	24.4 (9.3)	<.001
GARS	32.9 (14.3)	27.0 (8.7)	39.9 (16.4)	<.001
Stroop	82.7 (34.3)	78.8 (32.0)	92.2 (37.9)	<.001
Immediate WLT	17.0 (7.1)	18.3 (7.0)	13.6 (6.3)	<.001
Delayed WLT	8.7 (2.7)	9.2 (2.2)	7.5 (3.3)	<.001

^*^ *P* was estimated by the Chi-square test for categorical parameters, one-way ANOVA for normally distributed parameters and the Kruskal–Wallis test for non-normally distributed parameters.

ADL, activities of daily life; GARS, Groningen activities restriction scale; GDS, geriatric depression scale; IADL, instrumented activities of daily life; MMSE, mini mental state exam; WLT, word learning test.

Range: MMSE (0–30), GDS-15 (0–15) and GARS (18–72).

### Undirected analysis

**Figure 2:**
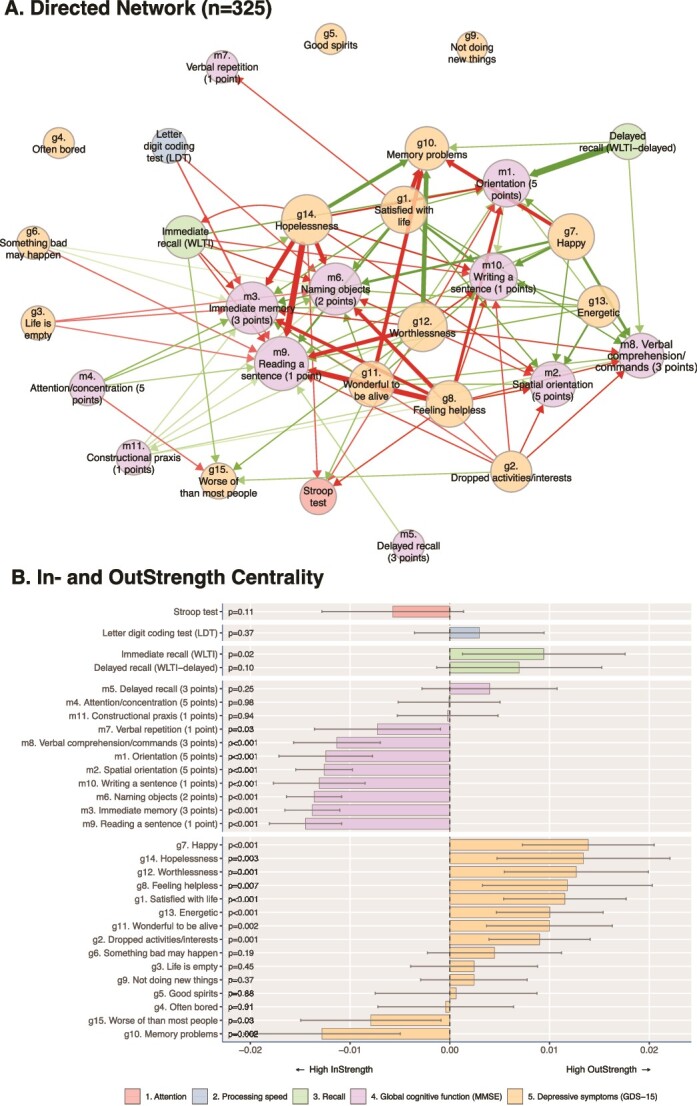
Undirected DTW analysis. (A) A nomothetic (i.e. applying to the group level) analysis of the undirected network plot based on panel data from 325 participants. Edges represent significant covariation in time (*P* < .001), with green edges denoting positive covariance over time, while red edges denoting negative covariance. (B) A dendrogram based on the Ward’s (D2, i.e. general agglomerative hierarchical clustering procedure) clustering of the 30 variables in the 325 distance matrices. For the full undirected distance matrix see Table S1 on page 2 of the Supplementary Materials.

The undirected DTW analysis of the 325 participants is shown in Figure 2 (for the undirected distance matrix, see Supplementary Materials, p. 2). Remarkably, the MMSE and GDS-15 did not exhibit distinct clustering patterns but showed intricate relationships. Focusing solely on the GDS-15, results showed a separate clustering, meaning the tendency to move up or down together in time, of apathy symptoms (‘energetic’, ‘dropped activities’ and ‘not doing new things’) and a separate clustering of mood symptoms (‘life is empty’, ‘something bad may happen’, ‘happy’, ‘satisfaction with life’, ‘wonderful to be alive’, ‘worthlessness’, ‘hopelessness’ and ‘feeling helpless’). ‘Memory problems’ of the GDS-15 were notably clustered with the mood-related symptoms. Another distinct cluster could be found, consisting of pessimism-related symptoms of the GDS-15 (‘good spirits’ and ‘worse off than most people’) and the more fundamental memory constituents of the MMSE (i.e. ‘reading a sentence’, ‘immediate memory’ and ‘naming objects’).

Furthermore, apathy, as assessed by the GDS-15, and the word learning tests were distinctively associated with cognitive aspects represented by the remaining MMSE constituents reflecting general cognitive function.

The Stroop test and the Letter Digit Coding Test showed minimal connections with other variables, suggesting that these test results showed rather independent trajectories within the broader network of variables being analysed.

### Directed analysis

**Figure 3:**
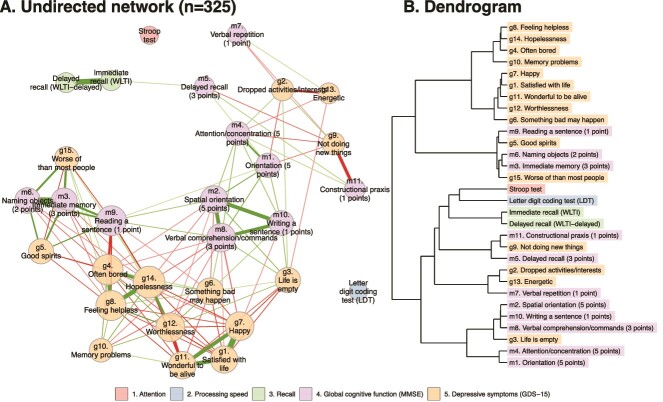
Directed DTW analyses. (A) Group level directed analysis of symptom clusters with symptoms network based on the average distance matrix of all 325 individual distance matrices. Arrows represent significant time-lagged relationships (*P* < .001). (B) In- and outstrength centrality measures the total incoming connections or influence that a node has in a network, indicating whether changes precede those of other symptoms and components, or whether changes follow those of other symptoms and components. For the full directed distance matrix see Table S2 on page 3 of the Supplementary Materials.

The directed DTW analysis is shown in Figure 3A (for the directed distance matrix, see Supplementary Materials, p. 3). The results display that the memory constituents of the MMSE were positioned very centrally and contained the most incoming arrows. This indicated that many deteriorations in the other, mostly depression and vitality symptoms, tended to precede changes (i.e. deterioration) in these MMSE constituents. Figure 3B demonstrates the extensive difference between the GDS-15 and MMSE constituents regarding the in- and outstrength centrality. Most of the MMSE constituents showed significant instrength centrality, suggesting them to be influenced by preceding changes in many of the nodes of the network. Notably, the more fundamental memory constituents of the MMSE, alongside the GDS-15 item 10 of ‘memory problems’, exhibited the highest instrength (*P* < .01). On the contrary, most of the GDS-15 symptoms, but especially the apathy symptoms, along with classical mood symptoms, such as hopelessness and worthlessness, showed significant outstrength centrality (*P* < .01). This indicated the many outgoing connections or influences that these nodes had on other nodes in the network. When employing a time window of 2 (years) instead of 1, we found largely consistent findings, albeit less statistically significant, with the GDS items (except g15 and g10) showing a higher outstrength centrality and the MMSE constituents showing a higher instrength centrality (data not shown). Finally, the WLTI showed significant outstrength, indicating a decline in these scores compared to other constituents of cognitive function. The WLTI-delayed, although not significant, showed a similar level of instrength. The Stroop test, the LDT, as well as some MMSE and GDS-15 symptoms, did not show significant in- or outstrength centrality (relative to the broader network of variables being analysed) and therefore did not have significant time relationships within the network.

## Discussion

In this study, population of the oldest old using the DTW-directed network analysis, we showed that within individuals, depressive symptoms preceded subsequent cognitive impairment. Moreover, depressive symptoms and cognitive impairment were found to be highly interconnected processes. The memory constituents of the MMSE had high instrength values, indicating that many changes in the other, mainly depressive, symptoms tended to precede changes in these core memory constituents. Notably, the only GDS-15 symptom demonstrating a significant instrength was ‘memory problems’, a symptom that captures subjective cognitive decline. These findings are indicative of Granger causality, which does not imply a direct cause-and-effect relationship but rather assesses whether one variable provides useful information for predicting another variable’s future values, establishing a statistical associations [31].

Regarding the network and cluster analyses on undirected DTW analyses, we found evidence of a cluster of apathy symptoms (consisting of items ‘dropped activities/interests’, ‘not doing new things’ and ‘energetic’) and mood symptoms (‘life is empty’, ‘something bad may happen’, ‘happy’, ‘satisfaction with life’, ‘wonderful to be alive’, ‘worthlessness’, ‘hopelessness’ and ‘feeling helpless’). This clustering is in line with the cross-finding of the ‘GDS 3A’ (apathy) subscale [32]. Furthermore, apathy symptoms were clustered with cognitive aspects of specific MMSE constituents, indicative of more subtle forms of cognitive impairment.

Our findings that depressive symptoms preceded cognitive impairment in a population-based sample of the oldest old are in line with that of numerous cohort studies [5–10]. However, these studies made inferences that were not based on aggregates of the individual dynamics, while we could quantify the individual dynamics first before they were aggregated, a crucial approach when dealing with potential non-ergodic relationships [33]. Several explanations can be given for finding a temporal association between depressive symptoms and cognitive impairment. Depressive symptomatology can present with clinical features that mimic cognitive impairment or be a prodrome of dementia, serving as an early warning [34]. Depression may also precede cognitive impairment through pathophysiological mechanisms. One underlying neurobiological mechanism could be the serotonergic system underlying both cognitive impairment and depression [35]. Also, depression may lead to cognitive impairment through enhancement of the accumulation of beta-amyloid plaques and tau tangles [36] in the brain, immune dysregulation [37] or chronic stress and activation of the hypothalamic–pituitary–adrenal (HPA) axis (leading to hippocampal atrophy) [38]. Another explanation could be the reciprocal relationship depression has with cardiovascular risk factors, such as hypertension, smoking and inflammation, contributing to atherosclerotic changes with decreased blood flow to the brain, which could lead to cognitive impairment [39]. Finally, anticholinergic drugs, such as certain classes of antidepressants, have been associated with an increased risk of cognitive impairment [40]. Yet, the association between depression and cognitive impairment is likely influenced by a combination of the abovementioned biological and sociobehavioral mechanisms, rather than a single cause. This is supported by the network theory of complexity in psychopathology [33, 41]. The link could further be caused by shared common lifestyle risk factors [42], such as smoking, decreased physical activity, poor diet, social isolation [43], feelings of loneliness [44] or shared genetic vulnerabilities [45], and underlying neurobiological substrates, like inflammatory alterations and declining levels, and activities of brain-derived neurotrophic factors [46].

DTW is an innovative analytical technique to compare the trajectories of symptoms over time and to analyse time-lag relationships. Although we are not aware of previous studies on cognitive impairment using DTW, some previous studies have analysed the time-series of depression symptoms using DTW analyses in patients with depression [15, 19], bipolar disorders [18] and post-traumatic stress disorder [47]. DTW allowed us to analyse individual panel data before aggregating the results, mitigating issues related to non-ergodic data and capturing unique temporal effects that may vary between individuals and may not occur at the same rate across the entire sample [21]. Another strength is that the dataset comprised individuals aged 85 years with comprehensive and well-documented cognitive assessment tools over repeated measurements in a follow-up period of up to 5 years. There are also some limitations to discuss. First, there were a limited number of assessments conducted, ranging from 3 to 5. This restricted frequency might have hindered our ability to capture the full and more subtle dynamics of cognitive impairment and its relationship with depressive symptoms. Second, the assessments were conducted at yearly intervals, and therefore, we were unable to capture dynamics and relationships within shorter time frames. Third, the GDS-15 and MMSE employed in this study are relatively crude assessment tools, which may have resulted in a lack of nuance and granularity in measuring depressive symptoms and their impact on cognitive decline. Additionally, while our study observed strongly significant findings (*P* < .001) regarding the relationship between depressive symptoms and cognitive decline using the GDS-15, it is essential to acknowledge the limitations inherent in the use of this instrument. The GDS-15, while commonly used as a screening tool for depression, may not be optimally validated for assessing individual elements, such as worthlessness, hopelessness, low happiness, dropping activities/interests and low satisfaction with life. Therefore, caution should be exercised when interpreting the results pertaining to these specific aspects of depressive symptomatology. For more precise analyses, the crucial constructs presented as nodes should each ideally be measured with several items or validated questionnaires in future research.

Our research underscores the clinical relevance of recognising the link between depression and cognitive decline. As of now, there is no cure or readily available treatment for cognitive decline. Therefore, it is even more important to unravel the clinical course and potentially influencing factors. Recognising the link between depression and cognitive decline may allow healthcare professionals to identify individuals at higher risk of developing cognitive impairment or dementia more promptly. Such early detection paves the way for the initiation of lifestyle modifications and the development of timely interventions and antegraded care. Treatment strategies for depression, like psychoeducation, physical activity, psychological therapies and antidepressants, have been shown to be effective even in old age [48], and treating depression early may also have a positive impact on cognitive function and overall QoL [49]. Understanding this association may also lead to better support and resources for caregivers and policymakers to manage the challenges of these conditions, such as programmes that combine face-to-face coaching with tailored Web-based modules [50]. This proactive approach is particularly crucial when it is expected that between 2015 and 2050, the proportion of the world’s population over 60 years will nearly double from 12% to 22%, and these conditions will become ever more prevalent [51].

In summary, our time-lagged DTW-directed network analysis revealed that within individuals depressive symptoms precede constituents of cognitive impairment in older people in the community.

## Supplementary Material

aa-23-2183-File005_afae130

## Data Availability

Data available from the authors on request.
