# Supplementary material for: Temporal dynamics of depressive symptoms and cognitive decline in the oldest old: dynamic time warp analysis of the Leiden 85-plus study
Source: Age Ageing. 2024 Jul 2;53(7):afae130. doi: 10.1093/ageing/afae130 (PMC11217552; doi:10.1093/ageing/afae130)
Supplement: aa-23-2183-File005_afae130 [file aa-23-2183-file005_afae130.pdf]

Supplementary Materials

This appendix has been provided by the authors to give readers addiDonal information about their work.

Supplement to: van der Slot AJC, Bertens AS, Trompet S, Mooijaart SP, Gussekloo J, van den Bos F, Giltay EJ. Temporal Dynamics of Depressive Symptoms and Cognitive Decline in the Oldest Old: Dynamic Time Warp analysis of the Leiden 85-plus Study.

Supplementary materials

Manuscript title: Temporal Dynamics of Depressive Symptoms and Cognitive Decline in the Oldest Old: Dynamic Time Warp analysis of the Leiden 85-plus Study.

Authors: van der Slot AJC, Bertens AS, Trompet S, Mooijaart SP, Gussekloo J, van den Bos F, Giltay EJ

Overview of supplementary tables: page

Table 1	Undirected network analysis distance matrix (n=325)	2
Table 2	Directed network analysis distance matrix (n=325)	3
DTW sample script (https://osf.io/wx8bk)		4
Correlation matrix		9
Undirected network analysis methods		10
Directed network analysis methods		11
MMSE		12
GDS-15		13

Undirected distance matrix (n=325)

	Stroop test	Letter digit coding test (LDT)	Immediate recall (WLTI)	Delayed recall (WLTI-delayed)	m1. Orientation (5 points)	m2. Spatial orientation (5 points)	m3. Immediate memory (3 points)	m4. Attention/concentration (5 points)	m5. Delayed recall (3 points)	m6. Naming objects (2 points)	m7. Verbal repetition (1 point)	m8. Verbal comprehension/commands (3	m9. Reading a sentence (1 point)	m10. Writing a sentence (1 points)	m11. Constructional praxis (1 points)	g1. Satisfied with life	g2. Dropped activities/interests	g3. Life is empty	g4. Often bored	g5. Good spirits	g6. Something bad may happen	g7. Happy	g8. Feeling helpless	g9. Not doing new things	g10. Memory problems	g11. Wonderful to be alive	g12. Worthlessness	g13. Energetic	g14. Hopelessness	g15. Worse of than most people
**Stroop test**		2.05	1.78	0.63	3.14**	1.38	4.09	2.50*	0.16	4.46	1.26	1.97*	1.50	1.29	0.07	4.66	-0.53	1.72	2.39	4.92	2.11	3.12	2.36	-1.30	1.89	4.59	2.15	1.08	2.03	6.31
**Letter digit coding test (LDT)**	2.05		2.75	3.18	1.27	2.21	8.03	0.86	2.39	8.40	2.06	2.07	5.03	2.68	1.39	6.57	2.79	4.43	6.02	8.80	4.30	5.21	5.84	1.46	6.43	7.57	5.17	3.03	4.85	9.85
**Immediate recall (WLTI)**	1.78	2.75		14.61***	3.08**	1.37	4.42	2.68**	3.61***	4.67	0.52	2.30*	1.56	1.46	0.35	3.20	-1.97*	1.02	2.96	5.91	2.63	1.77	2.99	-1.75	2.15	3.71	2.19	0.27	2.28	7.99
**Delayed recall (WLTI-delayed)**	0.63	3.18	14.61***		2.46*	-0.75	4.28	2.43*	2.75**	4.47	0.30	1.46	1.96	-0.65	0.48	3.66	-0.95	1.47	2.62	6.08	2.91	2.41	3.56	-1.43	2.17	4.25	2.55	0.93	2.55	6.90
**m1. Orientation (5 points)**	3.14**	1.27	3.08**	2.46*		8.27***	0.99	9.77***	3.50***	0.78	1.99*	7.22***	3.45***	6.23***	2.15*	0.30	-5.16***	-2.17*	-1.15	0.95	-1.81	0.86	-0.66	-4.47***	-0.31	1.07	-0.48	3.10**	-1.62	2.49
**m2. Spatial orientation (5 points)**	1.38	2.21	1.37	-0.75	8.27***		1.18	6.06***	0.05	0.79	0.58	12.41***	5.45***	12.41***	2.61	-2.87**	-2.54*	6.68***	-3.65***	0.16	-4.80***	-4.00***	-2.80**	0.29	-2.99**	-1.75	-3.42***	-1.29	-4.06***	1.77
**m3. Immediate memory (3 points)**	4.09	8.03	4.42	4.28	0.99	1.18		3.97	9.13	15.89***	10.22	0.67	10.36***	1.43	13.09	3.85	7.49	5.89	-5.46***	9.92***	1.05	3.57	-4.39***	9.01	-0.55	3.12	0.70	9.62	-4.33***	10.52***
**m4. Attention/concentration (5 points)**	2.50*	0.86	2.68**	2.43*	9.77***	6.06***	3.97		2.20*	3.56	1.38	6.07***	0.13	5.70***	4.02***	1.87	4.55***	-2.03*	1.18	4.40	-0.49	0.50	1.93	-5.07***	1.40	2.82	1.18	-3.58***	0.67	6.60
**m5. Delayed recall (3 points)**	0.16	2.39	3.61***	2.75**	3.50***	0.05	9.13	2.20*		8.28	1.22	1.38	4.63	0.08	4.61***	4.78	-2.54*	0.56	5.39	8.49	2.88	3.51	5.86	-4.73***	5.06	6.16	4.34	0.63	4.81	10.53
**m6. Naming objects (2 points)**	4.46	8.40	4.67	4.47	0.78	0.79	15.89***	3.56	8.28		10.50	0.20	9.93***	1.83	13.46	4.24	7.90	6.25	-5.10***	9.51***	1.38	3.96	-3.92***	9.32	-0.10	3.48	1.16	10.04	-3.89***	10.08***
**m7. Verbal repetition (1 point)**	1.26	2.06	0.52	0.30	1.99*	0.58	10.22	1.38	1.22	10.50		0.91	5.35	0.92	1.92	5.89	-3.64***	0.83	6.18	9.52	3.82	4.33	6.54	-3.30***	4.60	6.28	5.34	3.48***	5.15	11.90
**m8. Verbal comprehension/commands (3 points)**	1.97*	2.07	2.30*	1.46	7.22***	12.41***	0.67	6.07***	1.38	0.20	0.91		5.75***	11.84***	1.91	-3.37***	2.79**	5.47***	-3.63***	0.17	5.28***	-4.86***	-3.46***	0.86	-3.88***	-2.67**	-4.16***	-1.85	-4.62***	2.19
**m9. Reading a sentence (1 point)**	1.50	5.03	1.56	1.96	3.45***	5.45***	10.36***	0.13	4.63	9.93***	5.35	5.75***		3.27**	9.76	-0.59	4.19	2.14	-10.31***	9.03***	-3.50***	-0.77	-9.34***	6.25	-5.31***	-1.44	3.82***	5.78	-9.03***	-6.79***
**m10. Writing a sentence (1 points)**	1.29	2.68	1.46	-0.65	6.23***	12.41***	1.43	5.70***	0.08	1.83	0.92	11.84***	3.27**		2.96	-2.09*	3.26**	6.26***	-2.08*	1.91	4.65***	-3.80***	-1.03	0.08	-3.11**	-1.14	-2.61**	-1.48	-2.96**	4.25
**m11. Constructional praxis (1 points)**	0.07	1.39	0.35	0.48	2.15*	2.61	13.09	4.02***	4.61***	13.46	1.92	1.91	9.76	2.96		8.32	-3.23**	3.32	9.69	13.05	6.49	6.49	9.52	-11.56***	8.40	9.32	7.81	0.94	8.21	15.40
**g1. Satisfied with life**	4.66	6.57	3.20	3.66	0.30	-2.87**	3.85	1.87	4.78	4.24	5.89	-3.37***	-0.59	-2.09*	8.32		1.00	-6.52***	-5.36***	0.60	-6.83***	14.85***	-5.85***	5.95	2.63**	12.56***	-8.79***	1.44	-7.54***	3.08
**g2. Dropped activities/interests**	-0.53	2.79	-1.97*	-0.95	-5.16***	-2.54*	7.49	4.55***	-2.54*	7.90	-3.64***	2.79**	4.19	3.26**	-3.23**	1.00		4.42***	1.53	6.40	0.18	-0.66	2.05	4.77***	2.13	1.72	0.25	-10.28***	0.76	9.40
**g3. Life is empty**	1.72	4.43	1.02	1.47	-2.17*	6.68***	5.89	-2.03*	0.56	6.25	0.83	5.47***	2.14	6.26***	3.32	-6.52***	4.42***		2.64**	3.79	3.52***	-8.33***	1.54	1.01	0.16	-5.34***	5.29***	-3.93***	2.69**	6.26
**g4. Often bored**	2.39	6.02	2.96	2.62	-1.15	-3.65***	-5.46***	1.18	5.39	-5.10***	6.18	-3.63***	-10.31***	-2.08*	9.69	-5.36***	1.53	2.64**		-7.04***	4.33***	-4.31***	12.25***	6.77	5.54***	-6.23***	7.15***	4.03	11.68***	3.91***
**g5. Good spirits**	4.92	8.80	5.91	6.08	0.95	0.16	9.92***	4.40	8.49	9.51***	9.52	0.17	9.03***	1.91	13.05	0.60	6.40	3.79	-7.04***		-0.72	0.39	-5.79***	9.71	-1.61	0.30	-1.58	7.99	-5.69***	-7.34***
**g6. Something bad may happen**	2.11	4.30	2.63	2.91	-1.81	-4.80***	1.05	-0.49	2.88	1.38	3.82	5.28***	-3.50***	4.65***	6.49	-6.83***	0.18	3.52***	4.33***	-0.72		-6.98***	4.70***	3.86	-5.07***	-5.44***	6.78***	1.85	5.73***	2.71
**g7. Happy**	3.12	5.21	1.77	2.41	0.86	-4.00***	3.57	0.50	3.51	3.96	4.33	-4.86***	-0.77	-3.80***	6.49	14.85***	-0.66	-8.33***	-4.31***	0.39	-6.98***		-5.93***	4.38	-2.81**	12.41***	-8.63***	1.03	-7.69***	3.40
**g8. Feeling helpless**	2.36	5.84	2.99	3.56	-0.66	-2.80**	-4.39***	1.93	5.86	-3.92***	6.54	-3.46***	-9.34***	-1.03	9.52	-5.85***	2.05	1.54	12.25***	-5.79***	4.70***	-5.93***		6.24	5.39***	-6.80***	8.97***	3.83	13.04***	4.02***
**g9. Not doing new things**	-1.30	1.46	-1.75	-1.43	-4.47***	0.29	9.01	-5.07***	-4.73***	9.32	-3.30***	0.86	6.25	0.08	-11.56***	5.95	4.77***	1.01	6.77	9.71	3.86	4.38	6.24		5.24	6.45	5.39	-3.20**	5.36	12.16
**g10. Memory problems**	1.89	6.43	2.15	2.17	-0.31	-2.99**	-0.55	1.40	5.06	-0.10	4.60	-3.88***	-5.31***	-3.11**	8.40	2.63**	2.13	0.16	5.54***	-1.61	-5.07***	-2.81**	5.39***	5.24		4.28***	6.03***	3.64	6.86***	0.03
**g11. Wonderful to be alive**	4.59	7.57	3.71	4.25	1.07	-1.75	3.12	2.82	6.16	3.48	6.28	-2.67**	-1.44	-1.14	9.32	12.56***	1.72	-5.34***	-6.23***	0.30	-5.44***	12.41***	-6.80***	6.45	4.28***		-10.86***	2.43	-8.90***	2.53
**g12. Worthlessness**	2.15	5.17	2.19	2.55	-0.48	-3.42***	0.70	1.18	4.34	1.16	5.34	-4.16***	3.82***	-2.61**	7.81	-8.79***	0.25	5.29***	7.15***	-1.58	6.78***	-8.63***	8.97***	5.39	6.03***	-10.86***		0.68	10.57***	0.33
**g13. Energetic**	1.08	3.03	0.27	0.93	3.10**	-1.29	9.62	-3.58***	0.63	10.04	3.48***	-1.85	5.78	-1.48	0.94	1.44	-10.28***	-3.93***	4.03	7.99	1.85	1.03	3.83	-3.20**	3.64	2.43	0.68		2.15	10.28
**g14. Hopelessness**	2.03	4.85	2.28	2.55	-1.62	-4.06***	-4.33***	0.67	4.81	-3.89***	5.15	-4.62***	-9.03***	-2.96**	8.21	-7.54***	0.76	2.69**	11.68***	-5.69***	5.73***	-7.69***	13.04***	5.36	6.86***	-8.90***	10.57***	2.15		4.09***
**g15. Worse of than most people**	6.31	9.85	7.99	6.90	2.49	1.77	10.52***	6.60	10.53	10.08***	11.90	2.19	-6.79***	4.25	15.40	3.08	9.40	6.26	3.91***	-7.34***	2.71	3.40	4.02***	12.16	0.03	2.53	0.33	10.28	4.09***	

Data are adjusted mean difference in distance between the pair of items compared to the distance between all other items
***: p<0.05; **: p<0.01; ***: p<0.001**

directed distance matrix (n=325)

To (arrowhead in directed network)

From (tail of the arrow in directed network)

	Stroop test	Letter digit coding test (LDT)	Immediate recall (WLTI)	Delayed recall (WLTI-delayed)	m1. Orientation (5 points)	m2. Spatial orientation (5 points)	m3. Immediate memory (3 points)	m4. Attention/concentration (5 points)	m5. Delayed recall (3 points)	m6. Naming objects (2 points)	m7. Verbal repetition (1 point)	m8. Verbal comprehension/commands (3	m9. Reading a sentence (1 point)	m10. Writing a sentence (1 points)	m11. Constructional praxis (1 points)	g1. Satisfied with life	g2. Dropped activities/interests	g3. Life is empty	g4. Often bored	g5. Good spirits	g6. Something bad may happen	g7. Happy	g8. Feeling helpless	g9. Not doing new things	g10. Memory problems	g11. Wonderful to be alive	g12. Worthlessness	g13. Energetic	g14. Hopelessness	g15. Worse of than most people
Stroop test		0.006	0.000	0.000	0.015*	0.009*	-0.007	0.000	0.005	-0.006	0.009*	0.003	-0.006	-0.010**	0.003	0.000	0.000	0.004	0.000	-0.004	0.002	0.000	0.000	-0.002	0.004	0.000	0.000	0.000	0.000	0.005
Letter digit coding test (LDT)	-0.005		0.006	0.000	-0.003	-0.009**	-0.017***	0.006	0.000	-0.017***	0.007	-0.007*	-0.017***	-0.008**	0.006	-0.003	0.000	0.004	0.007	-0.010*	0.006	0.000	0.001	-0.001	0.010*	-0.002	0.003	0.000	0.000	0.015**
Immediate recall (WLTI)	-0.012*	-0.002		-0.001	0.024***	0.013**	-0.022***	-0.005	0.018*	-0.021***	0.007	0.012**	-0.019***	-0.018***	0.007	-0.006	-0.001	0.010*	0.014**	-0.016**	0.014**	-0.004	0.007	-0.008	0.015**	-0.009*	0.006	-0.003	0.017***	0.017***
Delayed recall (WLTI-delayed)	0.011	-0.009	0.001		0.035***	0.013**	0.011*	-0.010*	0.017*	0.013**	0.013**	0.016***	0.009*	-0.009*	-0.007	-0.003	-0.001	0.007	0.011*	-0.006	0.008*	0.000	0.002	0.004	0.016***	-0.004	0.000	0.000	0.007	-0.007
m1. Orientation (5 points)	-0.005	0.000	0.000	0.000		0.011*	0.000	0.003	0.000	0.000	0.000	0.011**	0.000	0.012***	-0.002	0.000	0.000	0.000	0.000	0.000	0.000	0.000	0.000	0.000	-0.003	0.000	0.000	0.000	0.000	0.000
m2. Spatial orientation (5 points)	0.000	0.000	0.000	0.000	0.000		0.002	0.000	0.000	0.002	-0.005	0.005	0.004*	0.015**	0.000	0.000	0.000	0.000	0.000	0.000	0.000	0.000	0.000	0.000	-0.005	0.000	0.000	0.000	0.000	0.000
m3. Immediate memory (3 points)	0.010*	0.000	0.000	0.000	0.005	-0.003		0.000	0.000	0.000	0.000	0.000	0.000	0.002	0.000	0.000	0.000	0.000	0.000	0.000	0.000	0.000	0.000	0.000	-0.005	0.000	0.000	0.000	0.000	0.000
m4. Attention/concentration (5 points)	0.000	0.000	0.000	0.000	-0.003	0.005	0.019***		0.000	0.018***	-0.005	0.009*	0.014***	0.008*	-0.002	-0.001	0.000	0.002	-0.004	0.008	0.000	-0.001	0.000	0.000	-0.009	0.000	0.000	0.000	0.003	-0.016***
m5. Delayed recall (3 points)	-0.006	0.001	-0.003	-0.004	-0.007*	0.013**	0.012**	0.008		0.012**	-0.010**	0.013**	0.012***	0.009*	-0.004	0.003	0.002	0.005	-0.003	0.006	-0.006	0.000	0.000	0.004	-0.010*	-0.002	0.000	-0.004	0.002	0.007
m6. Naming objects (2 points)	0.008*	0.000	0.000	0.000	0.007	-0.004	0.001	0.000	0.000		0.000	0.000	0.000	0.002	0.000	0.000	0.000	0.000	0.000	0.000	0.000	0.000	0.000	0.000	-0.004	0.000	0.000	0.000	0.000	0.000
m7. Verbal repetition (1 point)	0.000	-0.001	0.000	-0.004	0.001	0.002	-0.005	0.000	0.000	-0.004		-0.005	-0.004	0.002	-0.003	0.000	0.000	0.000	-0.001	0.000	0.000	0.000	0.000	0.000	0.004	0.000	0.000	0.000	0.000	0.002
m8. Verbal comprehension/commands (3 points)	0.000	0.000	0.000	0.000	-0.003	-0.001	-0.003	0.000	0.000	-0.003	0.000		0.007*	-0.001	-0.002	0.000	0.000	0.000	0.000	0.000	0.000	0.000	0.000	0.000	-0.005	0.000	0.000	0.000	0.000	-0.001
m9. Reading a sentence (1 point)	0.004	0.000	0.000	0.000	-0.004	-0.003	0.000	0.000	0.000	0.000	0.000	0.000		0.002	0.000	0.000	0.000	0.000	0.000	0.000	0.000	0.000	0.000	0.000	-0.004	0.000	0.000	0.000	0.000	0.000
m10. Writing a sentence (1 points)	0.003	0.000	0.000	0.000	-0.003	-0.001	-0.002	0.000	0.000	-0.002	-0.001	0.000	-0.002		0.000	0.000	0.000	0.000	0.000	0.000	0.000	0.000	0.000	0.000	0.000	0.000	0.000	0.000	0.000	0.001
m11. Constructional praxis (1 points)	-0.005	-0.003	0.000	0.000	0.005	0.007***	0.009***	0.005	0.007	0.010***	0.006	0.011***	0.009***	-0.006*		0.000	0.000	-0.002	-0.005	0.004	-0.001	0.000	0.000	0.000	-0.010**	0.000	-0.001	0.000	0.000	-0.009**
g1. Satisfied with life	0.013**	0.007	0.007	0.006	0.020***	0.023***	0.022***	0.012**	-0.005	0.023***	-0.019***	0.024***	0.027***	0.027***	-0.004		0.002	0.000	-0.012	0.013*	-0.004	0.000	0.002	-0.004	-0.031***	0.004	-0.004	0.002	0.002	-0.012*
g2. Dropped activities/interests	0.008*	-0.007	0.000	0.000	-0.015**	-0.024***	-0.017***	-0.012*	-0.004	-0.017***	0.014	-0.025***	-0.019***	-0.019***	0.007	-0.002		0.009	0.008	-0.008	0.005	-0.002	0.007	0.008	0.017**	-0.002	0.003	0.002	0.000	0.015***
g3. Life is empty	-0.006	-0.002	-0.006	-0.005	-0.009*	-0.016**	-0.015***	-0.004	-0.005	-0.014***	0.005	-0.018**	-0.014***	-0.019**	0.004	-0.003	0.000		0.004	0.000	0.002	0.000	0.000	0.002	0.014*	0.000	-0.001	0.000	0.000	0.005
g4. Often bored	-0.005	-0.002	0.000	-0.004	-0.012**	-0.009*	-0.013**	0.001	0.000	-0.012**	0.007	-0.005	-0.013*	-0.014**	0.002	0.000	-0.001	0.000		-0.001	0.000	0.000	0.000	0.003	0.019*	0.000	0.000	0.002	0.000	0.012
g5. Good spirits	0.014**	0.004	0.010	0.004	0.015*	-0.007*	0.016*	-0.002	-0.003	0.015*	-0.007	0.006	0.013*	0.009	-0.006	0.000	0.001	0.001	0.002		0.000	0.000	0.000	-0.003	-0.021**	-0.001	0.001	-0.002	0.001	-0.010
g6. Something bad may happen	-0.011**	-0.009*	-0.001	-0.002	-0.012**	-0.010*	-0.011**	-0.002	0.003	-0.011**	0.014**	-0.011*	-0.015***	-0.017**	0.006*	0.000	0.000	-0.001	0.005	-0.012*		0.000	-0.002	0.007*	0.013*	0.002	-0.002	0.000	0.000	0.005
g7. Happy	0.013**	0.010*	0.012*	0.010	0.023***	0.024***	0.025***	0.010*	0.003	0.027***	-0.013**	0.028***	0.026***	0.028***	0.007*	-0.001	0.003	-0.007	-0.012*	0.015*	-0.008		0.003*	-0.010*	-0.030***	0.000	0.002	-0.004*	0.003*	-0.016**
g8. Feeling helpless	-0.021***	-0.008	-0.012*	-0.009	-0.028***	-0.025***	-0.029***	-0.007	-0.006	-0.030***	0.009	-0.021***	-0.033***	-0.021***	0.004	-0.004	-0.004*	0.009	0.014	-0.017*	0.009	0.000		0.006	0.019*	-0.008	0.000	0.002	-0.001	0.021**
g9. Not doing new things	0.005	0.003	0.000	0.000	-0.007	-0.006	-0.009**	-0.007	-0.005	-0.009**	-0.005	0.007*	-0.008**	0.006*	-0.003	0.004	-0.003	-0.004	-0.006	0.006	-0.003	0.001	-0.003		-0.010**	0.005	0.000	0.002	-0.002	0.005
g10. Memory problems	-0.005	0.000	0.000	-0.007	0.004	0.001	0.004*	0.000	0.000	0.002	-0.002	0.001	0.002*	-0.002	0.000	0.000	0.000	0.000	-0.001	0.000	0.000	0.000	-0.001	0.000		0.000	0.000	0.000	0.000	-0.004*
g11. Wonderful to be alive	0.017***	0.005	0.012*	0.011*	0.016***	0.019***	0.023***	0.008*	0.002	0.023***	-0.013**	0.017***	0.026***	0.022***	-0.003	0.000	0.003	-0.011	-0.013	0.015*	0.000	0.000	0.000	-0.007	-0.030***		0.001	-0.001	0.001	-0.011
g12. Worthlessness	-0.017***	-0.011*	-0.003	-0.004	-0.019***	-0.019***	-0.025***	-0.007	-0.005	-0.025***	0.013**	-0.020***	-0.031***	-0.026***	0.011***	0.000	-0.002	0.012	0.011	-0.011	0.011	0.000	0.007	0.005	0.032***	-0.002		0.001	0.006	0.021***
g13. Energetic	-0.006	0.015**	0.009	0.005	0.015***	0.026***	0.018***	0.014*	0.012**	0.018***	-0.013	0.024***	0.020***	0.025***	0.008*	0.000	-0.007	-0.007	-0.009	0.008	-0.007	0.006	-0.002	-0.009*	-0.013*	0.003	-0.001		0.000	-0.011*
g14. Hopelessness	-0.017***	-0.007	-0.019***	-0.018**	-0.026***	-0.024***	-0.031***	-0.010	-0.004	-0.031***	0.007	-0.018**	-0.035***	-0.026***	0.006*	0.000	0.008	0.014*	0.015	-0.009	0.006	-0.005	0.010	0.009*	0.030***	-0.003	0.000	-0.008		0.023**
g15. Worse of than most people	-0.008	0.000	0.000	0.000	-0.009	0.002	-0.003	0.000	0.000	-0.003	0.000	0.000	-0.003	-0.005	0.002	0.000	0.000	-0.001	0.000	0.000	0.000	0.000	0.000	0.000	0.009	0.000	0.000	0.000	-0.001	

Data are the mean directed distance compared to zero (a positive value implies that changes in the first item precede changes in the second item)
***: p<0.05; **: p<0.01; ***: p<0.001**

```
#########################################################################
# Dynamic Time Warp (DTW) analyses
#
# Simplified r-script accompanying the paper:
# Temporal Dynamics of Depressive Symptoms and Cognitive Decline in the Oldest Old: Dynamic Time Warp analysis of the Leiden 85-
plus Study
# Abe J.C. van der Slot1, Anne Suzanne Bertens1,2, Stella Trompet3,4 , Simon P. Mooijaart3,4, Jacobijn Gussekloo3,4, Frederiek
van den Bos3,4 Erik J. Giltay1,5,†
#
# Affiliations:
# 1. Department of Psychiatry, Leiden University Medical Centre, Leiden, The Netherlands
# 2. Mental Health Care Rivierduinen, Leiden, the Netherlands
# 3. Department of Internal Medicine, Section of Gerontology and Geriatrics, Leiden University Medical Centre 2333 ZA Leiden, the
Netherlands
# 4. LUMC Center for Medicine for Older People, Leiden University Medical Center, Leiden, The Netherlands
# 5. Health Campus The Hague, Department of Public Health & Primary Care, Leiden University Medical Center, The Netherlands
#
# Date: April 2024
# email: e.j.giltay@lumc.nl
#

# packages (first install packages through "install.packages()")

library(tidyverse)
library(dtw)
library(parallelDist)
library(qgraph)
library(reshape2)

#########################################################################
# panel data from 3 participants
# load the data from participant A (467)

participantA <- tibble(
  time = 1:6,
  `m1. Orientation (5 points)` = c(0.78, 0.78, 0.78, 0.78, 0.78, 0.15),
  `m2. Spatial orientation (5 points)` = c(0.54, 0.54, 0.54, 0.54, 0.54, 0.54),
  `m3. Immediate memory (3 points)` = c(0.19, 0.19, 0.19, 0.19, 0.19, 0.19),
  `m4. Attention/concentration (5 points)` = c(0.75, 0.75, 0.75, 0.75, 0.75, 0.75),
  `m5. Delayed recall (3 points)` = c(0.52, 1.35, 1.35, 1.35, 1.35, 1.35),
  `m6. Naming objects (2 points)` = c(0.19, 0.19, 0.19, 0.19, 0.19, 0.19),
  `m7. Verbal repetition (1 point)` = c(-0.65, -0.65, -0.65, -0.65, -0.65, -0.65),
  `m8. Verbal comprehension/commands (3 points)` = c(0.50, 0.50, 0.50, 0.50, 0.50, 0.50),
  `m9. Reading a sentence (1 point)` = c(0.30, 0.30, 0.30, 0.30, 0.30, 0.30),
  `m10. Writing a sentence (1 points)` = c(0.53, 0.53, 0.53, 0.53, 0.53, 0.53),
  `m11. Constructional praxis (1 points)` = c(1.03, 1.03, 1.03, 1.03, 1.03, 1.03),
  `g1. Satisfied with life` = c(-2.31, -2.31, -2.31, -2.31, -2.31, -2.31),
```

```
  `g2. Dropped activities/interests` = c(1.46, -0.68, 1.46, 1.46, 1.46, 1.46),
  `g3. Life is empty` = c(1.86, 1.86, 1.86, 1.86, 1.86, 1.86),
  `g4. Often bored` = c(3.04, -0.33, -0.33, 3.04, 3.04, -0.33),
  `g5. Good spirits` = c(-4.15, 0.24, 0.24, 0.24, 0.24, 0.24),
  `g6. Something bad may happen` = c(2.35, -0.43, -0.43, 2.35, 2.35, -0.43),
  `g7. Happy` = c(-2.22, -2.22, -2.22, -2.22, -2.22, -2.22),
  `g8. Feeling helpless` = c(3.02, 3.02, 3.02, 3.02, 3.02, 3.02),
  `g9. Not doing new things` = c(-0.97, 1.03, 1.03, 1.03, 1.03, 1.03),
  `g10. Memory problems` = c(-0.38, -0.38, -0.38, -0.38, -0.38, -0.38),
  `g11. Wonderful to be alive` = c(-2.44, -2.44, -2.44, -2.44, -2.44, 0.41),
  `g12. Worthlessness` = c(2.48, 2.48, 2.48, 2.48, 2.48, 2.48),
  `g13. Energetic` = c(-1.51, -1.51, -1.51, -1.51, -1.51, -1.51),
  `g14. Hopelessness` = c(2.86, -0.35, 2.86, 2.86, 2.86, 2.86),
  `g15. Worse of than most people` = c(4.70, -0.21, -0.21, -0.21, 4.70, -0.21))

# load the data from participant B (285)

participantB <- tibble(
  time = 1:6,
  `m1. Orientation (5 points)` = c(0.78, 0.15, 0.15, 0.78, 0.15, -0.47),
  `m2. Spatial orientation (5 points)` = c(0.54, 0.54, 0.54, 0.54, 0.54, 0.54),
  `m3. Immediate memory (3 points)` = c(0.19, 0.19, 0.19, 0.19, 0.19, 0.19),
  `m4. Attention/concentration (5 points)` = c(-0.40, 0.17, 0.17, 0.75, -0.98, 0.75),
  `m5. Delayed recall (3 points)` = c(-1.13, 0.52, -1.13, -1.13, -1.13, -1.13),
  `m6. Naming objects (2 points)` = c(0.19, 0.19, 0.19, 0.19, 0.19, 0.19),
  `m7. Verbal repetition (1 point)` = c(-0.65, -0.65, -0.65, -0.65, -0.65, -0.65),
  `m8. Verbal comprehension/commands (3 points)` = c(0.50, 0.50, 0.50, 0.50, 0.50, 0.50),
  `m9. Reading a sentence (1 point)` = c(0.30, 0.30, 0.30, 0.30, 0.30, 0.30),
  `m10. Writing a sentence (1 points)` = c(0.53, -1.90, 0.53, 0.53, 0.53, 0.53),
  `m11. Constructional praxis (1 points)` = c(1.03, -0.97, -0.97, 1.03, -0.97, -0.97),
  `g1. Satisfied with life` = c(0.43, 0.43, -2.31, -2.31, -2.31, -2.31),
  `g2. Dropped activities/interests` = c(-0.68, -0.68, -0.68, 1.46, 1.46, 1.46),
  `g3. Life is empty` = c(1.86, 1.86, 1.86, 1.86, 1.86, 1.86),
  `g4. Often bored` = c(3.04, -0.33, 3.04, -0.33, 3.04, 3.04),
  `g5. Good spirits` = c(0.24, 0.24, 0.24, 0.24, 0.24, 0.24),
  `g6. Something bad may happen` = c(-0.43, -0.43, -0.43, -0.43, -0.43, -0.43),
  `g7. Happy` = c(0.45, 0.45, -2.22, -2.22, -2.22, -2.22),
  `g8. Feeling helpless` = c(-0.33, -0.33, 3.02, -0.33, 3.02, 3.02),
  `g9. Not doing new things` = c(-0.97, -0.97, 1.03, 1.03, 1.03, -0.97),
  `g10. Memory problems` = c(-0.38, 2.61, -0.38, 2.61, -0.38, -0.38),
  `g11. Wonderful to be alive` = c(0.41, 0.41, -2.44, -2.44, -2.44, -2.44),
  `g12. Worthlessness` = c(-0.40, -0.40, -0.40, -0.40, 2.48, 2.48),
  `g13. Energetic` = c(0.66, 0.66, -1.51, 0.66, -1.51, -1.51),
  `g14. Hopelessness` = c(-0.35, -0.35, 2.86, 2.86, 2.86, 2.86),
  `g15. Worse of than most people` = c(-0.21, -0.21, 4.70, 4.70, 4.70, 4.70))

# load the data from participant C (223)
```

```
participantC <- tibble(
  time = 1:6,
  `m1. Orientation (5 points)` = c(0.78, 0.78, 0.78, 0.78, 0.78, 0.78),
  `m2. Spatial orientation (5 points)` = c(0.54, 0.54, 0.54, 0.54, 0.54, 0.54),
  `m3. Immediate memory (3 points)` = c(0.19, 0.19, 0.19, 0.19, 0.19, 0.19),
  `m4. Attention/concentration (5 points)` = c(0.75, 0.75, 0.17, 0.75, -0.40, -0.98),
  `m5. Delayed recall (3 points)` = c(1.35, 1.35, -1.13, 1.35, -1.13, -0.30),
  `m6. Naming objects (2 points)` = c(0.19, 0.19, 0.19, 0.19, 0.19, 0.19),
  `m7. Verbal repetition (1 point)` = c(-0.65, 1.55, -0.65, -0.65, -0.65, -0.65),
  `m8. Verbal comprehension/commands (3 points)` = c(0.50, 0.50, -0.86, 0.50, 0.50, 0.50),
  `m9. Reading a sentence (1 point)` = c(0.30, 0.30, 0.30, 0.30, 0.30, 0.30),
  `m10. Writing a sentence (1 points)` = c(-1.90, 0.53, 0.53, -1.90, 0.53, 0.53),
  `m11. Constructional praxis (1 points)` = c(-0.97, -0.97, -0.97, 1.03, -0.97, -0.97),
  `g1. Satisfied with life` = c(0.43, 0.43, 0.43, -2.31, -2.31, -2.31),
  `g2. Dropped activities/interests` = c(1.46, 1.46, 1.46, 1.46, 1.46, 1.46),
  `g3. Life is empty` = c(-0.54, 1.86, 1.86, 1.86, 1.86, 1.86),
  `g4. Often bored` = c(3.04, -0.33, -0.33, -0.33, -0.33, -0.33),
  `g5. Good spirits` = c(0.24, 0.24, 0.24, -4.15, 0.24, -4.15),
  `g6. Something bad may happen` = c(2.35, 2.35, 2.35, 2.35, -0.43, -0.43),
  `g7. Happy` = c(0.45, 0.45, -2.22, -2.22, -2.22, -2.22),
  `g8. Feeling helpless` = c(3.02, -0.33, 3.02, 3.02, -0.33, -0.33),
  `g9. Not doing new things` = c(-0.97, -0.97, -0.97, -0.97, 1.03, -0.97),
  `g10. Memory problems` = c(-0.38, -0.38, 2.61, -0.38, -0.38, -0.38),
  `g11. Wonderful to be alive` = c(0.41, 0.41, -2.44, -2.44, -2.44, -2.44),
  `g12. Worthlessness` = c(-0.40, -0.40, -0.40, 2.48, -0.40, 2.48),
  `g13. Energetic` = c(0.66, -1.51, 0.66, -1.51, 0.66, -1.51),
  `g14. Hopelessness` = c(-0.35, -0.35, -0.35, -0.35, -0.35, -0.35),
  `g15. Worse of than most people` = c(-0.21, -0.21, -0.21, 4.70, 4.70, -0.21))

########################################################################
# functions to calculate the 3 distance matrices from one dataframe

calc_pair_directed <- function(var1, var2, db, timewindow, inverse){
  vector1 <- db[[var1]]
  vector2 <- db[[var2]]
  if (inverse == TRUE) {vector2 <- -vector2}
  dtw(vector2, vector1, window.size = timewindow, step.pattern = symmetric2,
      distance.only = TRUE, window.type = function (iw, jw, window.size, ...)
      {return(iw - jw <= window.size & iw - jw >= 0) } ) %>% .$distance
}

calc_pair_undirected <- function(var1, var2, db, timewindow, inverse){
  vector1 <- db[[var1]]
  vector2 <- db[[var2]]
  if (inverse == TRUE) {vector2 <- -vector2}
  dtw(vector2, vector1, window.size = timewindow, step.pattern = symmetric2,
      distance.only = TRUE, window.type = "sakoechiba") %>% .$normalizedDistance
}
```

```
calc_dtw <- function (db, timewindow = 1) {
  no_interpolations <- 5  # number of interpolations between assessments
  db2 <- db %>% map(~approx(.x, n = nrow(db) * no_interpolations)) %>%
    transpose() %>% as.data.frame() %>% select(-contains("x.")) %>% setNames(names(db))
  pair_list_db <- crossing(Var1 = names(db), Var2 = names(db))
  distance_positive1 <- map2(pair_list_db$Var2, pair_list_db$Var1, calc_pair_directed, db2,
                        timewindow * no_interpolations, inverse = FALSE) %>% unlist()
  distance_positive2 <- pair_list_db %>% cbind(value = distance_positive1) %>% acast(Var1 ~ Var2)
  distance_positive3 <- (distance_positive2 - t(distance_positive2)) / (distance_positive2 + t(distance_positive2))
  distance_inverse1 <- map2(pair_list_db$Var2, pair_list_db$Var1, calc_pair_directed, db2,
                        timewindow * no_interpolations, inverse = TRUE) %>% unlist()
  distance_inverse2 <- pair_list_db %>% cbind(value = distance_inverse1) %>% acast(Var1 ~ Var2)
  distance_inverse3 <- (distance_inverse2 - t(distance_inverse2)) / (distance_inverse2 + t(distance_inverse2))
  distance_direct1 <- melt(distance_positive3) %>% cbind(inv = melt(distance_inverse3)$value)
  distance_positive <- distance_direct1 %>%
    mutate(value2 = ifelse(!is.na(value) & value > 0 & abs(inv) < value, value + inv,
                           ifelse(!is.na(value) & value < 0 & abs(inv) < abs(value), value + inv, 0))) %>%
    acast(Var1 ~ Var2, value.var = "value2")
  distance_inverse <- distance_direct1 %>%
    mutate(inv2 = ifelse(inv < 0 & abs(inv) > abs(value), value + inv,
                         ifelse(inv > 0 & abs(inv) > abs(value), value + inv, 0))) %>%
    acast(Var1 ~ Var2, value.var = "inv2")
  distance_undir1 <- map2(pair_list_db$Var2, pair_list_db$Var1, calc_pair_undirected, db2,
                          timewindow * no_interpolations, inverse = FALSE) %>% unlist()
  distance_undir2 <- map2(pair_list_db$Var2, pair_list_db$Var1, calc_pair_undirected, db2,
                          timewindow * no_interpolations, inverse = TRUE) %>% unlist()
  distance_undirected <- pair_list_db %>% cbind(value = ifelse(distance_undir2 >= distance_undir1,
                                                           distance_undir1, -distance_undir2)) %>%
    acast(Var1 ~ Var2, value.var = "value")
  return(list(distance_undirected, distance_positive, distance_inverse))
}


########################################################################
# distance calculation with its distance matrices for participant A

distanceA <- calc_dtw(participantA[-1])

# undirected network plot and dendrogram for participant A

qgraph(1/distanceA[[1]], layout = "spring",
       label.prop = 3, label.scale.equal = T, labels = rownames(distanceA[[1]]), threshold = 3)

dist(distanceA[[1]]) %>% hclust() %>% plot()

# directed network plot for participant A
```

```
melt(distanceA[[2]]) %>% rename(value_pos = value) %>%
  left_join(melt(distanceA[[3]]) %>% rename(value_neg = value)) %>%
  mutate(value = ifelse(value_pos > value_neg, value_pos, -value_neg)) %>%
  acast(Var1 ~ Var2, value.var = "value") %>%
  qgraph(layout = "spring", label.prop = 3, label.scale.equal = T, labels = rownames(distanceA[[1]]), threshold = .1)

########################################################################
# distance calculation with its distance matrices for participant B

distanceB <- calc_dtw(participantB[-1])

# undirected network plot and dendrogram for participant B

qgraph(1/distanceB[[1]], layout = "spring",
       label.prop = 3, label.scale.equal = T, labels = rownames(distanceB[[1]]), threshold = 4)

dist(distanceB[[1]]) %>% hclust() %>% plot()

# directed network plot for participant B

melt(distanceB[[2]]) %>% rename(value_pos = value) %>%
  left_join(melt(distanceB[[3]]) %>% rename(value_neg = value)) %>%
  mutate(value = ifelse(value_pos > value_neg, value_pos, -value_neg)) %>%
  acast(Var1 ~ Var2, value.var = "value") %>%
  qgraph(layout = "spring", label.prop = 3, label.scale.equal = T, labels = rownames(distanceB[[1]]), threshold = .1)

########################################################################
# distance calculation with its distance matrices for participant C

distanceC <- calc_dtw(participantC[-1])

# undirected network plot and dendrogram for participant C

qgraph(1/distanceC[[1]], layout = "spring",
       label.prop = 3, label.scale.equal = T, labels = rownames(distanceC[[1]]), threshold = 4)

dist(distanceC[[1]]) %>% hclust() %>% plot()

# directed network plot for participant C

melt(distanceC[[2]]) %>% rename(value_pos = value) %>%
  left_join(melt(distanceC[[3]]) %>% rename(value_neg = value)) %>%
  mutate(value = ifelse(value_pos > value_neg, value_pos, -value_neg)) %>%
  acast(Var1 ~ Var2, value.var = "value") %>%
  qgraph(layout = "spring", label.prop = 3, label.scale.equal = T, labels = rownames(distanceC[[1]]), threshold = .1)

########################################################################
```

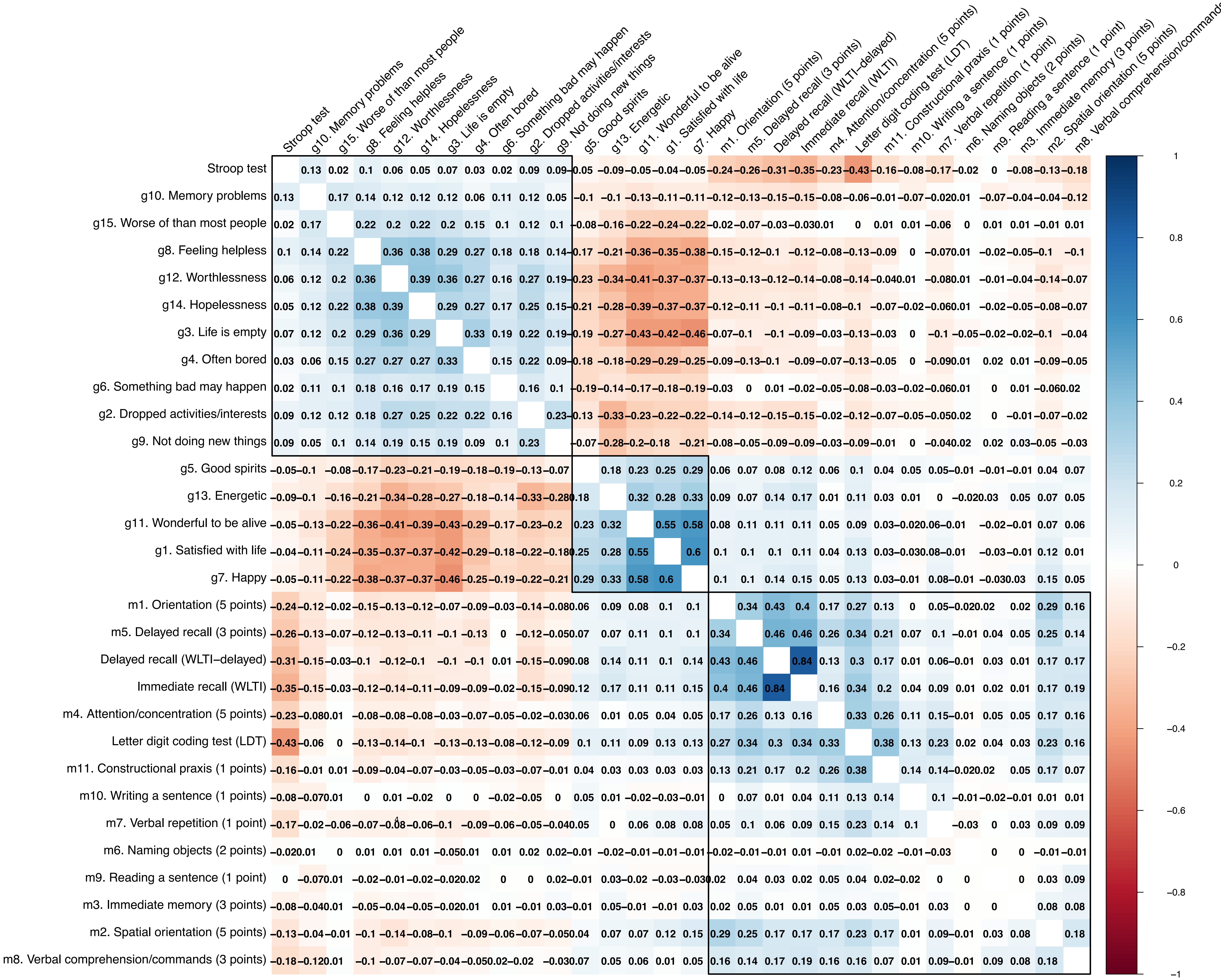
Stroop test
g10. Memory problems
g15. Worse of than most people
g8. Feeling helpless
g12. Worthlessness
g14. Hopelessness
g3. Life is empty
g4. Often bored
g6. Something bad may happen
g2. Dropped activities/interests
g9. Not doing new things
g5. Good spirits
g13. Energetic
g11. Wonderful to be alive
g1. Satisfied with life
g7. Happy
m1. Orientation (5 points)
m5. Delayed recall (3 points)
Delayed recall (WLTI–delayed)
Immediate recall (WLTI)
m4. Attention/concentration (5 points)
Letter digit coding test (LDT)
m11. Constructional praxis (1 points)
m10. Writing a sentence (1 points)
m7. Verbal repetition (1 point)
m6. Naming objects (2 points)
m9. Reading a sentence (1 point)
m3. Immediate memory (3 points)
m2. Spatial orientation (5 points)
m8. Verbal comprehension/commands (3 points)
1
0.8
0.6
0.4
0.2
0
−0.2
−0.4
−0.6
−0.8
−1

Undirected network analysis methods

In undirected analyses, DTW generated a distance matrix for each participant, representing the distances between each of the 30 by 30 variable pairs (e.g. comprising 11 MMSE sub scores, and 15 GDS item scores, Stroop test, letter digit coding test (LDT), immediate recall (WLTI), delayed recall (WLTI-delayed)). It resulted in an equal distance from variable 1 to variable 2 as from variable 2 to variable 1 (i.e., symmetric distance matrix). A lower distance indicated more similar dynamics, while a higher distance indicated more dissimilar trajectories, thus more independent changes over time. We set the time window at 1, meaning that scores of variable 1 could be stretched to match similar changes of variables 2 between t−1, t, and t + 1 (i.e., through a 'Sakoe-Chiba' time-window band of 1). To mitigate the potential disruptive effects of starting and endpoint mismatches, we applied interpolation with 5 values between each time point before calculating the distance between each variable pair.

We considered the presence of both positive (e.g., an increase is followed by an increase) and negative (e.g., an increase is followed by a decrease) relationships between variables. For each pair of variables, we computed two distances: one representing the distance from variable 1 to variable 2, and another representing the distance from variable 1 to the inverse of variable 2. For example, we would expect that a lower score on feeling energetic (GDS 13) would be associated with a higher score on dropping activities/interests (GDS 2). To determine the final distance between these variables, we selected the smallest absolute value among these two distances.

A hierarchical clustering method, specifically the Ward's minimum variance method ('ward.D2'), was employed to categorize the data based on similarities in their patterns. This technique was applied to the group-level distance matrix that was used to construct the undirected network plot. The reciprocal of the absolute values of the distances was used to effectively convert the distances to values, with higher original distances (indicating lower similarity) resulting in lower values. Ward's method then aggregated the items into clusters in a way that minimizes the total within-cluster variance, aiming to create more homogenous clusters. The undirected symptom network plot visualized the dynamic correlations or interactions between the 30 variables. In this plot the thickness of the edges corresponded to the strength of association and reflected the distances that were significantly smaller than the average of all remaining distances, after adjusting for the standard deviation scores of both items over time for each of the participants. A stringent P-value threshold of less than 0.001 was set for statistical significance, to account for multiple testing. This adjustment accounted for the clustering tendency of scores that remained zero or constant throughout follow-up to cluster more strongly together.

Directed network analysis methods

For the directed analyses of variables, we employed the same DTW algorithm as in the undirected analyses, with one important distinction. In this case, we specified an asymmetric window type (Figure 1). This choice ensured that information flow (i.e., stretching or warping) was assessed in one direction, specifically from variable 1 to variable 2, and not the other way around. We calculated a directed distance matrix for the standardized scores of the 30 variables for each of the 325 participants.

Similar to the undirected analysis, we assessed distances both representing the distance from variable 1 to variable 2, and that representing the distance from variable 1 to the inverse of variable 2. We subtracted the distance from variable 1 to the inverse of variable 2 from the distance from variable 1 to variable 2. If the resulting distance was zero, it indicated that there is no direct time-lagged influence from variable 1 to variable 2 (resulting in no arrow). When the resulting distance was greater than zero, it indicated a positive time-lagged effect from variable 1 to variable 2, represented by a green arrow. Conversely, if the resulting distance was smaller than zero, it indicated a negative time-lagged effect from variable 1 to variable 2 (i.e., when variable 1 increased, then variable 2 tended to decrease in the following time point, and vice versa), represented by a red arrow.

From the resulting group-level directed network plot, we averaged all distances for all pairs of variables. To determine if these averaged distances were significantly different from zero, t-tests were employed (with a threshold $P<0.001$). Significant directed edges in the plot were represented by arrows, with their tips pointing in the temporal direction, which is essential for establishing a (Granger causal) predictive relationship [1]. Out-strength centrality pertains to the number and strengths of outgoing edges originating from a particular node. In our DTW analysis, a high out-strength score for a variable implies that changes in that item tend to precede changes in other variable scores. On the other hand, in-strength centrality relates to the number and strengths of incoming edges to a specific node. In our DTW analysis, a high in-strength score for a variable suggests that its changes tend to follow changes in other variable scores. Descriptive analyses were done with SPSS version 25 (IBM Corp Released 2017, IBM SPSS Statistics for Windows, Version 25). Network analyses were done with the packages "dtw" (version 1.23–1) and "qgraph" (version 1.9.5) for the R statistical software (R version 4.3.1; R Foundation for Statistical Computing, Vienna, Austria, 2016. URL:https://www.R-project.org/).

References:

1. Granger, C.W.J., *Investigating Causal Relations by Econometric Models and Cross-spectral Methods.* Econometrica, 1969. **37**(3): p. 424–438.

The MMSE items include:

1. Temporal orientation (5 points): assessment of a person's awareness of time.
2. Spatial orientation (5 points): assessment of a person's awareness of place.
3. Immediate memory (3 points): a series of three unrelated words and that need to be repeated.
4. Attention/concentration (5 points): subtracting 7 from 100 and subsequent subtractions, which also tests calculation and working memory.
5. Delayed recall (3 points): recall the three words they were previously given.
6. Naming objects (2 points): language, naming two common objects.
7. Verbal repetition (1 point): repeat a phrase.
8. Verbal comprehension/commands (3 points): a multi-step instruction to test Verbal comprehension, commands, and executive function: "Take this paper in your right hand, fold it in half, and place it on the knee."
9. Reading a sentence (1 point): to assess the ability to understand and accurately pronounce the sentence.
10. Writing a sentence (1 points): to evaluates the individual's ability to generate a grammatically correct and meaningful sentence.
11. Constructional praxis (1 points): copy a drawing of intersecting geometric shapes.

The GDS-15 items include:

1. Are you basically satisfied with your life? YES / NO
2. Have you dropped many of your activities and interests? YES / NO
3. Do you feel that your life is empty? YES / NO
4. Do you often get bored? YES / NO
5. Are you in good spirits most of the time? YES / NO
6. Are you afraid that something bad is going to happen to you? YES / NO
7. Do you feel happy most of the time? YES / NO
8. Do you often feel helpless? YES / NO
9. Do you prefer to stay at home, rather than going out and doing new things? YES / NO
10. Do you feel you have more problems with memory than most people? YES / NO
11. Do you think it is wonderful to be alive? YES / NO
12. Do you feel pretty worthless the way you are now? YES / NO
13. Do you feel full of energy? YES / NO
14. Do you feel that your situation is hopeless? YES / NO
15. Do you think that most people are better off than you are? YES / NO
